# Adderall Induced Acute Liver Injury: A Rare Case and Review of the Literature

**DOI:** 10.1155/2013/902892

**Published:** 2013-06-23

**Authors:** Rohini R. Vanga, Bikram Bal, Kevin W. Olden

**Affiliations:** ^1^Beth Israel Deaconess Medical Center, Division of Gastroenterology, 330 Brookline Avenue, Dana 603, Boston, MA 02215, USA; ^2^Washington Hospital Center, Division of Gastroenterology, 110 Irving Street, Washington, DC 20010, USA; ^3^St. Joseph's Hospital and Medical Center, Division of Gastroenterology, 500 W Thomas Road, Suite 900A, Phoenix, AZ 85013, USA

## Abstract

Adderall (dextroamphetamine/amphetamine) is a widely prescribed medicine for the treatment of attention-deficit/hyperactivity disorder (ADHD) and is considered safe with due precautions. Use of prescribed Adderall without intention to overdose as a cause of acute liver injury is extremely rare, and to our knowledge no cases have been reported in the English literature. Amphetamine is an ingredient of recreational drugs such as Ecstacy and is known to cause hepatotoxicity. We describe here the case of a 55-year-old woman who developed acute liver failure during the treatment of ADHD with Adderall. She presented to the emergency room with worsening abdominal pain, malaise, and jaundice requiring hospitalization. She had a past history of partial hepatic resection secondary to metastasis from colon cancer which was under remission at the time of presentation. She recovered after intensive monitoring and conservative management. Adderall should be used carefully in individuals with underlying liver conditions.

## 1. Introduction

Acute hepatitis can result from wide variety of causes, among which viral and toxin induced injuries are the most common. Toxin induced liver injury contributes to 30% of acute liver injury [1]. Acetaminophen overdose is the most common cause of acute liver failure accounting for 39% of cases in United States [2]. Amphetamines and their derivatives are among the rarest causes of idiosyncratic drug reactions which are the presumptive cause in 13% of cases overall [2]. Amphetamine and Ecstasy (MDMA) remain popular recreational drugs in the western world but less common in United States. In Spain it is the second most common cause of acute hepatitis in patients under the age of 25 years, with viral hepatitis as the commonest cause [3]. Presentation can vary from subclinical elevation of liver enzymes to fulminant hepatic failure demanding orthotopic liver transplant [4]. It is extremely rare to develop liver toxicity at prescribed doses. To our knowledge, only one case has been reported in United States in a 14-year-old boy [5]. We report here the first case of Adderall induced acute liver injury in a 55-year-old female.

Approval from Institutional Review Board was obtained for case report submission.

## 2. Case Report

A 55-year-old female presented to Washington Hospital Center with three days history of malaise, anorexia, nausea, vomiting, jaundice, intense pruritus, and upper abdominal pain. She denied fever, chills, weight loss, and diarrhea. She also denied alcohol abuse, illicit drug use, and use of herbal supplementation. Her past medical history consisted of hypertension, hypothyroidism, Roux-En-Y Gastric bypass, ADHD, and colorectal cancer. Patient was diagnosed with stage IV (T3, M1, N1) malignant neoplasm of the ascending colon in 2002 for which she underwent right hemicolectomy. She had a partial hepatic resection (segments 4 and 7) in 2005 secondary to liver metastasis from colon cancer. Her colon cancer was in remission at the time of presentation. She has been on Adderall 30 mg twice a day for about eleven months. Patient took twice the recommended dose by herself (due to worsening of ADHD) for 5 days before she presented to the hospital. Her other medications included aspirin 81 mg, carvedilol 12.5 mg, and synthroid 50 mcg. Family history was negative for chronic liver disease including Wilson's disease, *α*-1-antitrypsin deficiency, and autoimmune hepatitis.

**Figure 1:**
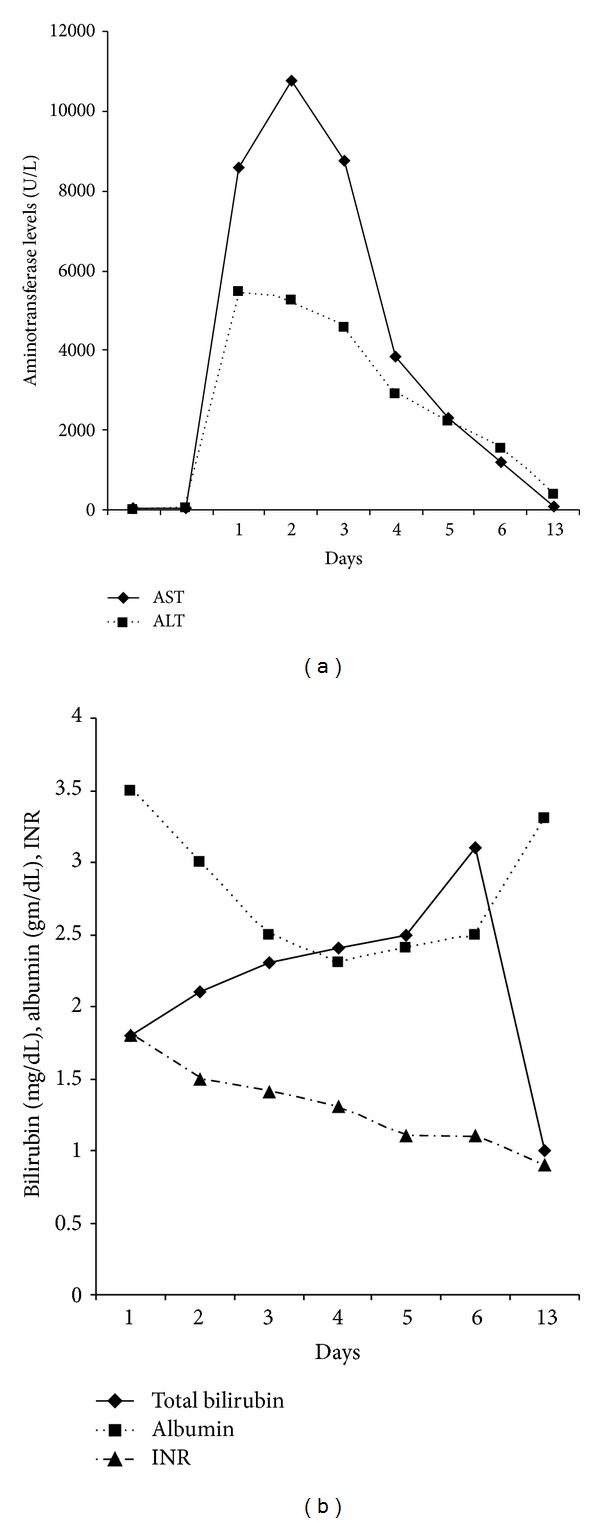
Biochemical markers of hepatocellular injury.

Patient remained hemodynamically stable for first 24 hours. Her hepatocellular injury was confirmed with biochemical markers. Additional investigation included serologic testing for cytomegalovirus, Epstein-Barr virus, and hepatitis A, B, and C viruses; results of all the serologies were negative for current or past infection. Her condition deteriorated by worsening encephalopathy, worsening of liver enzymes, and acute kidney injury by second day. Further evaluation also included testing her levels of acetaminophen, ceruloplasmin, *α*-1-antitrypsin level, antinuclear antibody, anti-liver/kidney microsomal antibody, anti-smooth-muscle antibody, amylase, and lipase. Imaging of the liver and biliary system was unremarkable. Evidence of hepatocellular and biliary injury was shown in her liver-panel results. In Figures 1(a) and 1(b), patient's bilirubin was elevated, serum albumin levels were low, and prothrombin time was elevated. After 72 hours of admission, patient's encephalopathy improved with lactulose, liver enzymes started trending down, and coagulation profile normalized. Renal function also improved with aggressive intravenous hydration. Given the remarkable improvement in her overall health condition, liver biopsy was deferred at that point. Patient was discharged on the seventh hospital day and continued to do well upon followup in the clinic a week after discharge. At 3-month followup, her aminotransferase levels were AST of 25 U/L and ALT of 22 U/L (normal <40 U/L as per the laboratory standard).

We arrived at a diagnosis of Adderall induced acute liver injury after an extensive evaluation for viral, metabolic, and autoimmune conditions that failed to reveal a cause for hepatitis in this patient. Her clinical presentation, with symptoms emerging after administration of the drug and cessation of symptoms shortly after withdrawing the drug, led to our conclusion that the hepatitis resulted from a reaction to Adderall. According to the Naranjo scale, it is probable (score = 5) that this case of acute liver failure was a result of an adverse drug reaction [6]. We also applied the modified Council for International Organizations of Medical Sciences (CIOMS) criteria for determining drug-related hepatotoxicity to our case [7]. The CIOMS criteria include a rechallenge with the suspect medication, which we did not deem ethical. However, a probable score (score = 6) was attained even without the potential points for a rechallenge.

## 3. Discussion

Our case is the first case in the medical literature that has resulted in enormous elevation of transaminases due to amphetamine toxicity. Various mechanisms of amphetamine and its derivatives causing liver injury have been mentioned in the literature [8, 9]. Lack of cytochrome P450 oxidase CYP2D6 in 5%–9% of Caucasians results in accumulation of methoxyamphetamine and hydroxyamphetamine causing hepatocyte damage [8]. Immune-mediated mechanisms have been hypothesized to play a part in amphetamine induced liver damage [8]. Hyperthermia induced hepatocyte oxidative damage by lipid peroxidation remains a significant etiological possibility [10]. Since amphetamine has actions similar to cocaine [11], ischemic injury has been implicated but there was no evidence of it in rat models [12]. Cocaine induced hepatotoxicity can lead to zonal and periportal coagulative necrosis with both macrovesicular and microvesicular fatty changes in residual hepatocytes [12]. Amphetamine induced hepatotoxicity may variously manifest at a histological level as a microvesicular fatty change, small foci of cell necrosis or massive hepatic necrosis. There is no evidence of hemodynamic alterations to liver blood flow in amphetamine or ecstasy intoxication [8].

Since amphetamines are the rare cause of acute liver injury, physicians should first exclude common etiology of acute hepatic failure which includes acetaminophen overdose, viral hepatitis, autoimmune causes, Wilson's disease, hemochromatosis, and portal and hepatic vein thrombosis. The ingestion/presentation interval and short half-life of the drug frequently lead to a negative result in blood or urine. It is crucial to monitor the serial prothrombin time estimations, serum bilirubin, transaminases, and albumin levels. Liver biopsy should be considered if the extent of liver damage or the etiology is in doubt (should be carried out by transjugular route if PT is significantly prolonged). Accurate clinical assessment of renal function and adequate hydration is also required. Hyperthermia should be treated aggressively. Drug induced acute liver failure is considered to have worse clinical outcomes [13]. Patients should be considered for emergency liver transplantation on individual basis if the conservative measures fail. However, data on the survival rate after liver transplantation in amphetamine induced fulminant hepatic failure is limited.

## 4. Conclusion

Although few cases of Ecstasy and Amphetamine induced acute liver injury have been reported in the medical literature, no case of Adderall induced acute liver injury has been reported. In our patient, hepatic resection may have resulted in compromised functional reserve which, in turn, might have led to Adderall induced hepatic insult. Meticulous supportive care was crucial for our patient with compromised liver function. Clinicians need to be alert to possible liver injury when using Adderall especially in a similar setting.
